# The impact of multidimensional physical activity feedback on healthcare practitioners and patients

**DOI:** 10.3399/bjgpopen18X101628

**Published:** 2019-02-06

**Authors:** Max J Western, Dylan Thompson, Oliver J Peacock, Afroditi Stathi

**Affiliations:** 1 Lecturer in Behavioural Science, Department for Health, University of Bath, Bath, UK; 2 Professor, Department for Health, University of Bath, Bath, UK; 3 Lecturer in Environmental Physiology, Department for Health, University of Bath, Bath, UK; 4 Reader, School of Sport, Exercise & Rehabilitation Sciences, University of Birmingham, Edgbaston, UK

**Keywords:** Physical Activity, Fitness Trackers, Feedback, Qualitative Research, Emotions, Primary Health Care

## Abstract

**Background:**

Promotion of physical activity in primary care has had limited success. Wearable technology presents an opportunity to support healthcare practitioners (HCPs) in providing personalised feedback to their patients.

**Aim:**

To explore the differing thoughts and feelings of both HCPs and at-risk patients provided with personalised multidimensional physical activity feedback.

**Design & setting:**

Qualitative study with HCPs (*n* = 15) and patients at risk of cardiovascular disease or type 2 diabetes (*n* = 29), recruited from primary care.

**Method:**

HCPs and patients wore a physical activity monitor for 7 days and were subsequently shown their personalised multidimensional feedback, including sedentary time, calorie burn, short (1-minute) or long (>10-minute) bouts of moderate-to-vigorous activity during semi-structured interviews. Transcripts were analysed thematically with comparisons made between individuals of high (*n* = 21) and low (*n* = 23) physical activity levels as to their cognitive–affective responses to their data.

**Results:**

Personalised feedback elicited positive emotional responses for highly active participants and negative emotional responses for those with low activity. However, individuals with low activity demonstrated largely positive coping mechanisms. Some low active participants were in denial over feedback, but the majority valued it as an opportunity to think of ways to improve physical activity (cognitive reappraisal) and started forming action plans (problem-focused coping). Around half of all participants also sought to validate their feedback against peers.

**Conclusion:**

Personalised, visual feedback elicits immediate emotional and coping responses in participants of high and low physical activity levels. Further studies should explore whether multidimensional feedback could help practitioners explore diverse ways for lifestyle change with patients.

## How this fits in

Primary care could be a useful setting for promoting physical activity, but short consultation times and a lack of GP knowledge or skills make it challenging. Little is known on how wearable technology could aid practitioners in promoting active lifestyle with their patients. The benefits of physical activity can be achieved in a variety of ways. Personalised, multidimensional, visual feedback provides a motivational environment for lifestyle evaluation. Patients of all levels of physical activity respond well to this feedback, which could provide an important tool for a successful lifestyle consultation in primary care.

## Introduction

Regular engagement in physical activity can substantially reduce the risk of numerous chronic diseases.^1^ Unfortunately, not enough people meet the recommended levels of physical activity and the prevalence of conditions such as cardiovascular disease and type 2 diabetes is increasing.^2,3^ GPs can play an important role in the promotion of physical activity to patients who are at risk of such conditions by educating them on recommended levels, and helping them develop strategies to increase their daily activity.^4^ However, time pressures (due to short doctor–patient consultations) and a lack of GP knowledge or confidence in promoting physical activity make providing suitable advice challenging.^5,6^ Innovative ways to support GPs in providing their patients with clear, motivating guidance are therefore required.

Wearable monitoring technology has markedly improved our understanding of the protective properties of physical activity in recent years.^7^ As these devices become more sophisticated, there is an opportunity to capture information in relation to different types of physical activity, allowing healthcare professionals to explore a range of potential areas that patients feel more confident to try to change.^8^ Examples of these distinct behavioural components include an increase in the time spent in light, moderate, or vigorous activity, a reduction in the amount of time spent sedentary, and an increase in total energy expenditure.^9^ In theory, presenting several aspects of behaviour to patients empowers them with more ways to change that align with their habits, interests, needs, and personal goals.^10^ HCPs could therefore use multidimensional feedback to frame short discussions with patients around the appropriateness of their existing physical activity, motivate them to self-monitor, and set personalised behavioural goals.^11^

For wearable devices to facilitate an effective behaviour change, the feedback they provide to a patient needs to be easily understood and trusted, and to support patients to take action towards personal health goals.^12^ The authors recently demonstrated that multidimensional physical activity feedback is comprehendible and useful for practitioners and patients, but the psychological responses to such feedback has yet to be explored.^13^ Prominent theories of health behaviour change, such as the Health Action Process Approach^14^ and the Common Sense Model of Illness representation,^15^ propagate the role that barriers, attitudes, risk perception, emotions, coping, and action plans play on behaviour. The way users handle affective response to feedback — be it avoidance or denial, problem solving, coping, cognitive reappraisal, or social support seeking — is likely to influence the subsequent actions they take, guiding practitioners on how much additional support their patients might need.^16^ The emotional and cognitive responses are also likely to differ based on whether the feedback conveys a positive or negative message, as well as the experience a patient has with regards to physical activity, and the importance they place on it.^17^

The aim of this study was to explore the affective and cognitive responses to personalised multidimensional visual physical activity feedback, and highlight necessary skills that practitioners need to further develop in order to manage the responses of their patients.

## Method

### Study design

This study presents a content analysis of qualitative interviews held with HCPs and patients, collected during the development phase of the Mi-PACT randomised controlled trial.^18^ This study was conducted in three stages: the first stage involved the development of physical activity graphics that appropriately depicted multiple health harnessing aspects of physical activity. The second phase involved all participants wearing a physical activity monitor for 1 week in order to generate personal feedback, before a final stage in which participants were shown their feedback as part of a one-to-one semi-structured interview.

#### Participants

A total of 44 participants from south west England were interviewed. Of these, 29 were patients identified as being at moderate (10.0–19.9%) or high (>20.0%) risk of cardiovascular disease and/or type 2 diabetes via a GP database search and 15 were regional HCPs, including GPs (*n* = 3), nurses (*n* = 4), healthcare assistants (*n* = 2), and exercise referral trainers (*n* = 6) who had experience working with such patients. Physical activity status and other demographic information is displayed in Table 1.

**Table 1:** Descriptive characteristics of all participants included in the analyses

Characteristic	Low activity group, *n* (%) (*n* = 23)	High activity group, *n* (%) (*n* = 21)	*P* value^a^
Patient, *n*	15	14	
HCP, *n*	8	7	
**Sex**			NS^b^
Male	12 (52)	15 (71)	
Female	11 (48)	6 (29)	
**Mean age, years**	58.6 (8.9)	56.9 (12.6)	NS
Patient mean age, years (range)	61.7 (44–70)	63.6 (50–71)	NS
HCP mean age, years (range)	52.8 (41–66)	43.6 (31–61)	NS
**Marital status**			NS^b^
Single/ widowed/ divorced	9 (39)	6 (29)	
Married/ cohabiting	14 (61)	15 (71)	
**Highest educational attainment**			NS^b^
Up to A-Level or equivalent	10 (43)	8 (38)	
First degree or higher degree	13 (57)	13 (62)	
Mean height, years (SD)	1.72 (0.11)	1.76 (0.08)	NS
Mean weight, kg (SD)	81.1 (16.7)	79.2 (14.0)	NS
Mean BMI, kg/m^2^ (SD)	27.2 (4.2)	25.6 (3.8)	NS
Mean waist circumference, cm (SD)	92.6 (12.6)	90.6 (13.4)	NS
**Physical activity dimension**			
Mean physical activity level, PAL ratio (SD)^c^	1.66 (0.25)	1.96 (0.26)	<0.001
Mean sedentary time, % waking day (SD)^d^	75.8 (7.3)	61.8 (10.1)	<0.001
Mean daily moderate activity, minutes (SD)^e^	84.1 (30.9)	171.3 (63.9)	<0.001
Mean MVPA bouts, minutes/week (SD)^f^	240.4 (148.2)	643.3 (327.9)	<0.001
Mean vigorous activity, minutes/week (SD)^g^	40.7 (55.6)	182.7 (166.4)	<0.001

^a^Differences between groups tested using independent *t*-test unless specified. ^b^Tested using Pearson χ^2^ test for proportional differences. ^c^Physical activity level: average total daily energy expenditure/basal metabolic rate (kcal/day). ^d^Sedentary time: percentage of waking day spent under 1.5 METs (480 minutes of sleep was assumed and subtracted from the total). ^e^Daily moderate activity: average number of single minutes of moderate activity accumulated in 24 hours (≥3 METs, <6 METs). ^f^MVPA bouts: all activity greater than 3 METs sustained for at least a period of 10 minutes and accumulated across the week. ^g^Vigorous activity: all the minutes of vigorous activity (>6 METs) accumulated over the monitored week.

METs = metabolic equivelent of task. MVPA = moderate to vigorous intensity physical activity. HCP = healthcare professional. NS = not significantly different between groups. PAL = physical activity level.

#### Feedback

**Figure 1.**
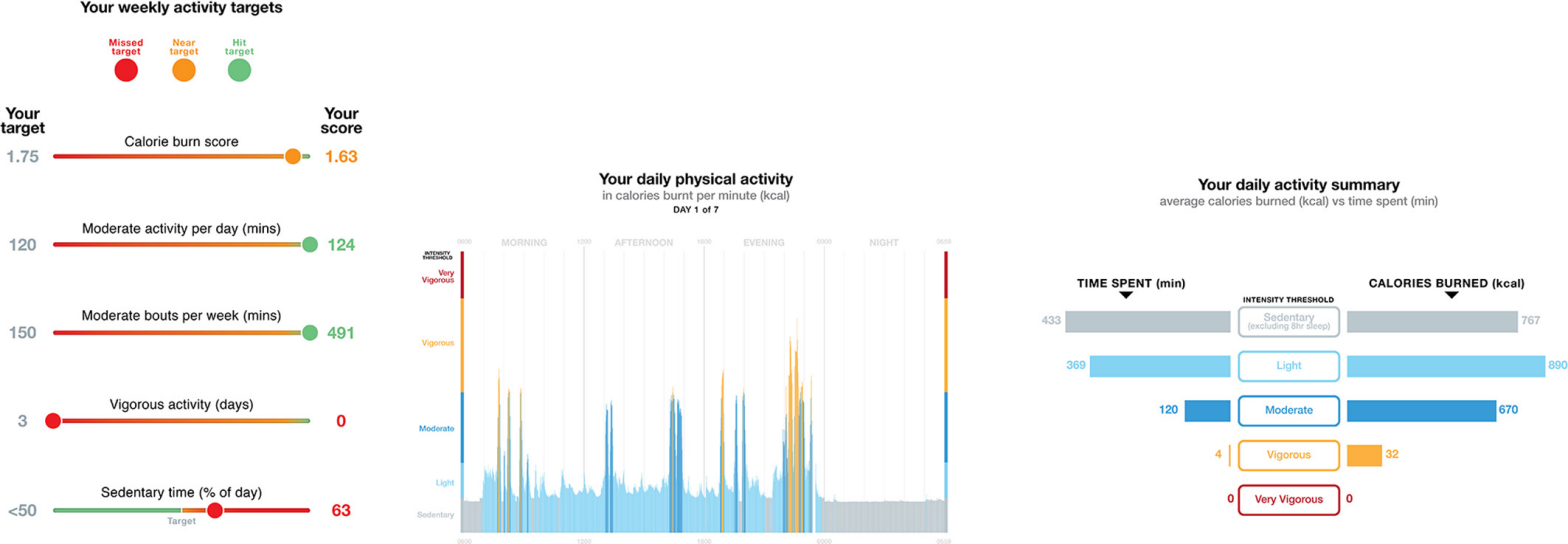
Examples of personalised feedback shown to participants. From left to right: health target attainment represented by traffic light colours across the five important health dimensions in a sliding scale format; a single day activity pattern colour graded by activity intensity; summary bar charts depicting the proportion of a given day spent in each intensity threshold and its resulting calorie expenditure

Multidimensional visual feedback included information on an individual’s performance in relation to five independent physical activity dimensions, including physical activity level (target = 1.75 x resting energy expenditure [kcal/day]); average sedentary time (target = <60% waking day); time engaged in moderate activity accumulated on a minute-by-minute basis (target = 120 minutes/day); moderate-to-vigorous physical activity accumulated in at least sustained 10-minute bouts (target = 150 minutes/week); and total vigorous activity time (target = 75 minutes/week).^8^ A traffic light system indicated whether the participant had hit the target (green), was near (amber, ≤ 25% below the target), or missed (red, >25% below target). Participants were also shown detailed visual summary statistics of the average time per day spent in each intensity threshold, distinguished using a temperature colour scale (Figure 1). Participants were classified as ‘low active’ (*n* = 23) if they met none, one, or two of the presented health recommendations, and ‘high active’ (*n* = 21) if they met three, four, or all five of the health targets. The scores in all physical activity dimensions were significantly different between high and low activity groups (*P*<0.001).

#### Procedure

To generate the feedback each participant was provided with an arm-mounted Bodymedia Mini Armband (SenseWear Pro 8.0) at a meeting in which they signed informed consent and had their height, weight, and other demographic details measured. Participants were instructed to wear the device for 1 ‘typical’ week and asked to only remove it for showering or water-based activities.^19^ Personalised feedback was then created for each participant and presented to participants during a one-to-one interview with the lead researcher. Interviews took place either at the workplace for HCPs or their GP practice, or University of Bath for patients and lasted ≥2 hours. Topic guides were compiled with input from an expert panel of academics and health practitioners and were split into three sections.

Section one included questions to gauge the participant’s thoughts on physical activity (for example, meaning, current levels, and barriers to and motivators for engagement) and was asked prior to any feedback being shown. Section two included an appraisal of the different visual representations of their personalised multidimensional feedback in terms of clarity, and immediate thoughts and feelings resulting from that feedback (for example, 'How does seeing your personalised data make you feel?'). The interviewer introduced each set of graphics before showing the participants their own data, but did not aid the interpretation of the graphics themselves. Section three explored the potential application of these graphics in supporting patients to make lifestyle changes, and elicited further responses based on the overall thoughts and feelings the participant had shared during the consultation.

#### Analysis

Interviews were digitally recorded and audio files transcribed verbatim and then uploaded to NVivo (Version 9.0) for coding and data organisation. A thematic content analysis approach^20^ was used to analyse the data. An initial coding frame designed to identify responses relating to a) attitudes, barriers, and any general thoughts on physical activity; and b) the feelings and thoughts experienced upon receiving feedback. Inductive themes that emerged from the data were recorded. The accuracy of the themes was confirmed by one member of the research team and agreed by other members of the research team. Once all transcripts were fully coded and checked, the research team looked at between-subject patterns in order to classify common recurring viewpoints, and distinguished the views of individuals of high or low physical activity levels.

## Results

Three key overarching themes emerged including attitudes towards physical activity (prior to receiving any feedback), the emotional responses to feedback, and coping mechanisms. The subthemes under each of these headings are described in the subsequent paragraphs with reference to any differences between individuals classed as 'high active' (HA) or 'low active' (LA). The activity group, role (patient [PAT], or healthcare professional [HCP]), sex, and age of participants are presented next to the quotes.

### Attitudes towards physical activity prior to receiving feedback

The attitudes of both patients and HCPs differed according to their personal activity levels. HA participants tended to value physical activity as important for their health and wellbeing, and most had a good handle on their activity levels prior to receiving feedback:

*'I am convinced that exercise has an effect on all parts of my body. Um, this is my muscles and bones, my wellbeing, my brain and the way it works, and my cardiovascular and respiratory systems.'* (HA, PAT, female, 70)

*'It’s something that I practice, as well as preach haha… as I’ve got older I’ve really understood the benefits with regards to health, the actual health side of things.'* (HA, HCP, female, 31)

Fewer LA participants were forthcoming in suggesting physical activity was important compared to HA participants. Those who did profess that they thought it important would often do so in the context of a personal barrier:

*'Probably…I mean, I suppose I could do more, I would like to do more, they’re probably not sufficient really but my back is weak but I suppose I ought to make more effort I probably don’t do enough.'* (LA, PAT, male, 65)

*'So I do go to the gym, and I go quite a lot. I go most days apart from the days that I work all day, you know when I do a long day, but even then I do sometimes go.'* (LA, HCP, female, 46)

When asked about the barriers to engaging in (more) physical activity, the HA individuals cited ageing and a physical inability to do more as their main restricting factor. LA participants on the other hand cited a lack of control through external environmental or psychosocial barriers such as lack of time, high cost, a dislike of gyms, or a lack of social support:

*'Uh, age and level of fitness stops me from going back and playing squash. Not so much age, level of fitness and level of training is stopping me from doing the more extreme walking under more difficult conditions. I tend to be very careful about, you know, how much an ascent and decent is, and been planned in my walks. I would like to be able to do more.'* (HA, PAT, male, 57)

*'My husband hates it, well he doesn’t hate it, but his idea of a walk is to the station and back… My friends who I used to do that with all moved away, one comes occasionally I suppose. It’s the lack of people to do it with I think, so that’s why I have to do* [walk on] t*he streets but it’s a bit boring.'* (LA, PAT, female, 65)

HCPs were asked to describe the barriers for a typical patient and a recurring opinion in those with high activity levels was that physical activity was not seen as important or a priority:

*'Because if your health is really important to you, the number one thing is gonna be being active, so it’s going to be a high priority to you. So people don’t perceive it as being that much of a priority and they kind of put it off.'* (HA, HCP, male, 37)

*'You know now it is something you have to take individual responsibility for and I think ... for some people you know, taking individual responsibility is absolutely beyond them, so ... probably that’s the single biggest barrier I would say.'* (HA, HCP, male, 45)

LA HCPs would generally cite barriers similar to those described by patients themselves including time, expense, dislike of gyms, low self-esteem, and low social support:

*'You know, "don’t have time", or … you know "the weather’s been horrible, I can’t get out for a walk because it’s been … " you know, "it’s been too cold, it’s been too wet", you know it’s always … it’s always been something else getting in the way.'* (LA, HCP, female, 51)

### Emotional response to feedback

Upon receiving their feedback, a large number of the LA interviewees expressed feelings of disappointment, shame, and guilt, and very few conveyed positive affect. These feelings were particularly apparent when interpreting the 24-hour activity pattern data (for example, seeing how sedentary one was being) and seeing that they had met so few of the five health targets. The expression of negative emotions was more apparent in individuals who hit none, as opposed to one or two, of the targets:

*'It makes me feel actually lazy. I have to admit that I’m ashamed of my lack of activity. It certainly brought it home to me, yes.'* (LA, PAT, male, 70)

*'Makes me feel I don’t do very much. It’s awful. And yet I feel, in my own way, that I’m quite busy doing things. But there you go ... it doesn’t look like it when I see all that round there'* (LA, PAT, female, 70)

HA participants on the other hand tended to express feelings of satisfaction, relief, and reassurance upon receiving their personal feedback. This was particularly evident when reviewing their health targets, although interestingly, almost half of the HA participants, including some who met four or all of the guidelines, did express some dissatisfaction when interpreting their activity patterns:

*'Oh that’s nice. Gosh. That seems as if it’s ... from my personal point of view I could be considered to be quite satisfied with that I imagine … Fine.'* (HA, PAT, female, 71)

*'Eight hours of sedentary time in the day feels shocking ... I probably have just never looked at this before but it's scary that I consider myself quite an active person now and there's still eight hours of sedentary time in there on average.'* (HA, PAT, male, 57)

HCPs reacted much in the same way as their patients when interpreting their own feedback:

*'A very high percentage of my day is ... hmm that’s not good, it’s making me feel bad.'* (LA, HCP, female, 52)

*'That is horrible. Because I would have hoped to be much more in the vigorous level. That is absolutely dreadful when you average it out. The only good thing is at least that’s hitting the health level, but that is so scary for somebody that does an hour in the gym each day.'* (LA, HCP, male, 58)

*'I’m sure relieved um, but it’s also providing some constructive insight into the best way to change … '* (HA, HCP, male, 45)

*'Well I think I probably do sufficient exercise to keep my weight stable. Um, very good actually. Yep. Hard work has paid off.'* (HA, HCP, female, 52)

### Coping mechanisms

Approximately half of the LA individuals appeared to use denial as an immediate coping mechanism, suggesting that the data presented might not have been a typical representation of their normal levels:

*'I probably would have done slightly more on the moderate side, but because um, it was the school holidays, and my daughter was away, I probably didn’t have to do quite as much … sort of running around and housework and general domestic duties that I would normally have to do.'* (LA, HCP, female, 49)

*'I’m sure that’s not a normal week. The moderate activity is far, far more and the vigorous activity is probably a little bit more because of the walking, which I haven’t done.'* (LA, PAT, female, 51)

That said, almost all LA HCPs and patients also appeared to rationally acknowledge and identify with a need to do more, irrespective of earlier views on their levels of physical activity and the importance they placed on it. This indicates a process of cognitive reappraisal in response to their emotional reaction:

*'Interesting. Gosh. I find that I'm spending almost — well, nine hours or four-fifths of my day doing sedentary activities. It's good that to see it because, you can't really avoid a few home truths so it's effective.'* (LA, PAT, male, 46)

*'Um … it’s interesting to see, I can’t believe that when you’re sedentary … how does that work? Anyway, it’s um … well, I look at that instantly and I think "right, I’ve got to do more … "’* (LA, HCP, female, 50)

Many of the LA participants also adopted an immediate problem-focused coping response, whereby they would either describe general behavioural goals in the context of their feedback or specific action plans towards adopting more physical activity:

*'The surprise factor is that in, in everyday life when I don’t try hard at it I am actually getting some light to moderate exercise. So if I tried harder at it I could probably boost it without too much effort. Yeah and presumably even if I walk around the block um, it’s okay it’s not going to take me 10 minutes but, but at least it’s 10 minutes of walking rather than 10 minutes of sat down watching television.'* (LA, PAT, male, 60)

*'It has and it’s ... as I just indicated, I need to understand my pattern of work and rest really to see, um, if I could increase, this a bit … I would look at that and think "well, actually, I’m not enough on my moderate, maybe I need a decent walk over the hill a bit more often". Because that could be perhaps moderate or depending if you’re walking pretty quick and over the hill that’d be classed as vigorous then wouldn’t it?'* (LA, PAT, male, 61)

HA patients were much less inclined to express any desire to change their behaviour, although those who had not achieved all health targets, or felt their activity pattern data to be disappointing, did state their intent to do so in the future. There was also a tendency amongst many HA and LA participants to seek out normative data against which to validate their feedback:

*'As I said before there's room for improvement. It does make me think you know more about keeping healthy, and doing more exercise, you know trying to do more exercise … I think I have to be less sedentary. Have to get up and walk about at work a lot more.'* (HA, PAT, female, 70)

*'Yeah. I just wonder in general it might be nice to have sort of ... what is an average line through the middle see if you’re above average, below average...'* (HA, PAT, male, 60)

*'If I hadn’t done that run there that would’ve been vigorous because I would’ve been panting. So what’s the average, how’s the general population doing, are they missing their targets?'* (LA, PAT, male, 44)

## Discussion

Personalised multidimensional visual physical activity feedback evoked an immediate emotional reaction in HCPs and patients. This could act as a catalyst for setting a meaningful agenda for behaviour change discussions in primary care consultations.^6^ The specific characteristics of individual patient’s feedback could help practitioners anticipate their emotional responses and tailor their consultation to promote effective coping mechanisms by their patients. For highly active patients, a positive ‘keep it up’ message and brief guidance on how they might overcome perceived physical limitations or environmental influences in order to achieve any missed targets, might be sufficient to support maintenance of or improvements to their existing behaviour. The LA patients on the other hand might need greater reassurance as they interpret their data. In particular, HCPs who are themselves highly active may need to detach themselves from potential preconceptions about patients’ attitudes, and show empathy in light of the barriers to physical activity that their patients face.^21^ A practitioner can also steer patients’ reflections about their lifestyle towards more specific, concrete action plans and specific lifestyle goals.^4,22^

### Strengths and limitations

Strengths of the present study include a) the objective assessment of physical activity made by a practical and reliable wearable device; and b) the recruitment of a diverse sample of HA and LA HCPs and patients, allowing a wide range of responses to be captured. The authors also presented a novel, multidimensional approach that included reference to light activity and short bouts of moderate activity that are often omitted from contemporary physical activity promotion but are featuring heavily in the new US technical report, which will inform the revisions of the physical activity guidelines in the US and UK.^23^

A limitation of the study is the cross-sectional design, which only captures participants’ responses at one point in time. While this gives good insight into how people respond to personalised visual data at first glance, it is not possible to conclude how stable these cognitive–affective responses would be over time, nor whether the action plans discussed by patients would lead to action. Furthermore, the interview in the present study, which served multiple purposes (for example, to rate different formats of feedback), was much longer than a typical GP–patient consultation. Longitudinal designs that investigate a HCP delivering the technology-enabled multidimensional feedback to their patients over single or multiple short consultations would be appropriate in examining how effective it could be in driving lifestyle changes in a more real life context.

### Comparison with existing literature

The desire for HA participants to seek out normative comparisons and focus on shortcomings rather than strengths in their personal data is consistent with the idea that more experienced individuals might respond better to specific, performance-based feedback (for example, meeting specific targets).^17^ Novices respond better to positive feedback as it reinforces their competence and commitment to a new goal.^15^ Results of the present study suggest that negative feedback to LA participants’ does lead to negative affect, which overtime may trigger negative evaluation of current lifestyle choices and loss of motivation.^24^ HCPs will therefore benefit from drawing on the positive coping responses and guide patients towards setting realistic, achievable goals and action plans.^11,25^

Positive feedback also directs the attention to problem-solving coping rather than avoidance or denial as this was expressed by a few participants who reported feelings of guilt or shame upon seeing their feedback. Support for emotional reactions, overcoming barriers, and formulating action plans could be key for helping patients initiate positive behaviour changes.^14,26^ The multidimensional approach used is especially useful for overcoming individual barriers and tailoring goals as the different health targets can be presented as distinct opportunities for change. For example, patients who cite physical limitations or lack of time as barriers to participation could concentrate on reducing the time spent sitting and use visual activity pattern data to identify opportunities to gradually build on existing, rather than adding new and therefore time-consuming, behaviours.^10^

Recent intervention studies that have employed physical activity feedback in primary care settings have shown to be effective for promoting and maintaining behavioural change.^27,28^ The PACE-UP and PACE-LIFT trial, which involved patient cohorts similar to the one in the present study, found that a 12-week pedometer or pedometer plus accelerometer feedback of activity intensity intervention, with and without nurse consultation, helped patients increase and sustain their daily steps by around 650 per day, and moderate to vigorous intensity physical activity (MVPA) by around 25 minutes per day over 3–4 year follow-up.^29^ Intervention participants in PACE-UP and PACE-LIFT were very positive about the use of digital feedback in a primary care setting with practitioner support, and reported similar facilitators and barriers to engagement in regular physical activity as participants in the present study.^28,30^ These studies also shed light on behaviour change techniques that might be key for the successful implementation of the novel, multidimensional format of feedback presented in the present study, such as self-monitoring, prompting goal review, planning social support and change, and relapse prevention. While physical activity technology can be used in isolation, other qualitative work in similar at-risk patient populations suggests that coach support is useful for sustained motivation and engagement.^31^

### Implications for practice

Health advocates are starting to recognise the multidimensional nature of physical activity, and the need to focus on substituting sedentary behaviour for light activity and de-emphasising the need for long, sustained activity bouts of moderate intensity.^32^ An understanding of how patients and practitioners think and feel about feedback in this format is therefore key to its operationalisation. Multidimensional feedback allows practitioners to explore diverse ways for lifestyle change with their patients while maintaining a positive climate, even with patients with low activity levels. The positive interpretation and cognitive–affective response suggests that such feedback may be a useful tool for practitioners with which to frame a brief, personalised discussion on physical activity with their patients and support setting specific, measurable, and achievable goals and action plans where required.

The successful adoption of technology in primary care ultimately rests on not burdening practices in terms of cost, GPs in terms of time, or patients in terms of it feeling clinical and impersonal.^33^ Fortunately, wearable devices for capturing physical activity behaviour are becoming ever more popular, accurate, and affordable, making it a more viable option for patients and healthcare professionals.^34^ Transforming objective physical activity data into meaningful visual information can be a quick, automated process.^18^ The fact that LA patients can respond positively to negative personalised feedback suggests that using such a tool could help motivate users into action.
